# Fertilization Induces a Transient Exposure of Phosphatidylserine in Mouse Eggs

**DOI:** 10.1371/journal.pone.0071995

**Published:** 2013-08-07

**Authors:** Claudio A. Curia, Juan I. Ernesto, Paula Stein, Dolores Busso, Richard M. Schultz, Patricia S. Cuasnicu, Débora J. Cohen

**Affiliations:** 1 Instituto de Biología y Medicina Experimental, Consejo Nacional de Investigaciones Científico y Técnicas, Buenos Aires, Argentina; 2 Department of Biology, University of Pennsylvania, Philadelphia, Pennsylvania, United States of America; 3 Department of Nutrition, Diabetes and Metabolism, Facultad de Medicina, Pontificia Universidad Católica de Chile, Santiago, Chile; Indiana University School of Medicine, United States of America

## Abstract

Phosphatidylserine (PS) is normally localized to the inner leaflet of the plasma membrane and the requirement of PS translocation to the outer leaflet in cellular processes other than apoptosis has been demonstrated recently. In this work we investigated the occurrence of PS mobilization in mouse eggs, which express flippase *Atp8a1* and scramblases *Plscr1* and *3*, as determined by RT-PCR; these enzyme are responsible for PS distribution in cell membranes. We find a dramatic increase in binding of flouresceinated-Annexin-V, which specifically binds to PS, following fertilization or parthenogenetic activation induced by SrCl_2_ treatment. This increase was not observed when eggs were first treated with BAPTA-AM, indicating that an increase in intracellular Ca^2+^ concentration was required for PS exposure. Fluorescence was observed over the entire egg surface with the exception of the regions overlying the meiotic spindle and sperm entry site. PS exposure was also observed in activated eggs obtained from CaMKIIγ null females, which are unable to exit metaphase II arrest despite displaying Ca^2+^ spikes. In contrast, PS exposure was not observed in TPEN-activated eggs, which exit metaphase II arrest in the absence of Ca^2+^ release. PS exposure was also observed when eggs were activated with ethanol but not with a Ca^2+^ ionophore, suggesting that the Ca^2+^ source and concentration are relevant for PS exposure. Last, treatment with cytochalasin D, which disrupts microfilaments, or jasplakinolide, which stabilizes microfilaments, prior to egg activation showed that PS externalization is an actin-dependent process. Thus, the Ca^2+^ rise during egg activation results in a transient exposure of PS in fertilized eggs that is not associated with apoptosis.

## Introduction

The anionic phospholipid phosphatidylserine (PS) is a relatively minor constituent of most biological membranes. Nevertheless, its unique physical and biochemical properties indicate that it is physiologically important [1]. PS is asymmetrically distributed in the plasma membrane, localizing almost exclusively in the inner leaflet of the lipid bilayer [2], and the loss of PS asymmetry is typically associated with the recognition and clearance of apoptotic cells [3]. Accumulating evidence, however, supports the idea that exposure of PS on the extracellular compartment of the membrane also plays essential roles in different cellular processes, including platelet pro-coagulant activity [4], host innate immune system evasion in obligate intracellular parasites such as *Leishmania mazonensis* and *Tripanosoma cruzii*, [5], trophoblastic intercellular fusion [6], enveloped vaccinia virus infection [7], and sperm capacitation [8],[9].

The distribution of PS within the plasma membrane is the result of the concerted action of two different Ca^2+^-regulated enzymes: ATP-dependent aminophospholipid translocases or flippases, and aminophospholipid scramblases [10]. Flippases actively catalyze mobilization of phospholipids to the inner leaflet of the lipid bilayer, and therefore are postulated as responsible for the asymmetric distribution of PS. On the other hand, scramblases translocate phospholipids in both directions of the plasma membrane, allowing exposure of PS in the outer membrane leaflet [11].

In mammals, fertilization is characterized by the generation in eggs of long-lasting Ca^2+^ oscillations, which initiate a series of changes collectively known as “egg activation”. These changes include early events such as cortical granule (CG) exocytosis that is involved in the prevention of polyspermy, and the resumption of the meiosis, as well as late events including extrusion of the second polar body, sperm head decondensation, and mRNA recruitment (see [12]). Although the role of Ca^2+^ in initiation of these events is clearly established, a complete description of all the molecular effectors involved still remains partially elucidated. The increasing evidence for a role of PS exposure in different non-apoptotic cellular events, together with the critical role that Ca^2+^ plays in both PS mobilization and fertilization, led us to explore PS mobilization in fertilized eggs. We report that PS becomes exposed after fertilization of mouse eggs and that PS exposure is transient. This exposure is not associated with apoptosis and does not require metaphase II exit or the presence of CaMKIIγ. The fertilization-induced PS exposure, however, requires both the increase in intracellular calcium ([Ca^2+^]_i_) and cytoskeleton remodelling that occur during egg activation.

## Materials and Methods

### Ethics statement

Animal experimental procedures were reviewed and approved by the Ethical Committee of IBYME (CE 003-1/2011), and the Institutional Animal Care and Use Committee (IACUC) of the University of Pennsylvania (protocol number 803766). Experiments were performed in strict accordance with the Guide for Care and Use of Laboratory Animals approved by the National Institutes of Health (NIH).

### Animals and reagents

Hybrid C57BL/6xCBA)F1 or CF1 adult (60–120 days) male and young adult (30–60 days) female mice, as well as young adult CaMKIIγ^−/−^ [13] female mice were used. Animals were maintained at 23°C with a 12 h L:12 h D cycle.

All reagents and chemicals were of molecular biology grade and were purchased from Sigma-Aldrich Chemicals (St Louis, MO, USA), or Invitrogen (Carlsbad, CA, USA), unless otherwise specified.

### *In vitro* sperm capacitation

Mouse sperm were recovered by incising the *cauda epididymides* in 300 µl of capacitation medium [14] supplemented with 0.3% of bovine serum albumin (BSA, Sigma) under paraffin oil (Ewe, Sanitas SA, Buenos Aires, Argentina). Aliquots of the suspension were added to 300 µl of fresh medium previously placed in tissue culture dishes to give a final concentration of 1–3×10^6^ cells/ml. Sperm suspensions were then incubated for 90 min under paraffin oil at 37°C in an atmosphere of 5% CO_2_ in air.

### Egg collection and *in vitro* fertilization

Female mice were superovulated by an injection (i.p.) of equine chorionic gonadotropin (eCG; 5 UI; Syntex SA, Buenos Aires, Argentina), followed by the administration (i.p.) of human chorionic gonadotropin (hCG; 5 UI, Sigma) 48 h later. Eggs were collected from the oviducts of superovulated animals 12–13 h after hCG administration. Cumulus cells were removed by incubating the cumulus-oocyte complexes for 5 min in 0.3 mg/ml hyaluronidase (type IV; Sigma). *Zona pellucidae* (ZP) were dissolved by treating the eggs with acid Tyrode's solution (pH 2.5) for 10–20 sec [15].

ZP-free eggs were inseminated with capacitated sperm (final concentration: 0.5×10^4^ cells/ml) and the gametes co-incubated for 1 to 24 h at 37°C in an atmosphere of 5% CO_2_ in air. For polyspermy assays, ZP-free eggs were inseminated with a higher volume of sperm to achieve a final concentration 1×10^4^ cells/ml, and the gametes co-incubated for 3 h. ZP-intact eggs were inseminated with capacitated sperm (final concentration 5×10^5^ sperm/ml) and the gametes were co-incubated for 3 h. Eggs were considered fertilized when at least one decondensing sperm nucleus or two pronuclei were observed in the egg cytoplasm after Hoechst staining (see below).

### Parthenogenetic egg activation

Metaphase II-arrested (MII) eggs were cultured in complete CZB medium [16] containing 7% ethanol for 5 min or 100 µM TPEN (Sigma) for 1 h. Alternatively, eggs were incubated in Ca^2+^/Mg^2+^-free CZB containing 10 mM SrCl_2_ (Sigma) for 1 h, or 5 µM A23187 Ca^2+^ ionophore (Sigma) for 5 min. In some cases, eggs were incubated with 50 µM BAPTA-AM (Molecular Probes, Life Technologies Co., USA) for 20 min to chelate intracellular calcium, and then activated with SrCl_2_ as described. For experiments with actin-perturbing drugs, eggs were incubated with 10 µM cytochalasin D (cytD, Sigma) or with 0.5 µM jasplakinolide (Jas, Invitrogen) for 60 min prior to activation with SrCl_2_ and during subsequent culture. In all cases, after activation, the eggs were transferred to CZB medium for further culture. Eggs were considered activated when re-initiation of meiosis was observed after Hoechst staining (see below).

### PS and DNA staining

To determine the presence of externalized PS, FITC-conjugated Annexin V (FITC-ANX5, 1∶25, BD Pharmingen, USA) was added during the last hour of gamete co-incubation or parthenogenetic egg activation. At the end of this incubation, the eggs were stained with 1 µg/µl Hoechst 33342 (Sigma), washed, mounted, and examined with a Nikon Optiphot microscope (Nikon, Tokyo, Japan) equipped with epifluorescence optics (250×). For quantification of ANX5 fluorescent labeling, FITC-ANX5-incubated fertilized and non-inseminated eggs were photographed and analyzed with ImageJ 1.42 q software (Waine Rasband National Institutes of Health, USA). The total surface fluorescence intensity/area, as well as the fluorescence intensity in the labeled areas, were calculated for fertilized eggs, and normalized to the values obtained for non-inseminated eggs.

For confocal microscopy, FITC-ANX5 incubated eggs were fixed in freshly prepared 3.7% p-formaldehyde in PBS for 20 min, washed in 0.1% BSA 0.01% Tween 20 in PBS, and mounted under a coverslip by gentle compression in a Vectashield (Vector Laboratories, Inc., USA) solution containing TO-PRO 3 (Life Technologies, USA) or Hoechst 33342. Fluorescence was detected on a Leica TCS SP or a Nikon D-Eclipse C1 (E800) laser scanning confocal microscope.

### Cortical granule and actin staining

For CG content staining, eggs were fixed for 60 min in freshly prepared 3.7% p-formaldehyde, washed 3 times in 0.3% BSA 0.1 M glycine in PBS, permeabilized with 0.1% Triton X-100 in 0.3% BSA-PBS (TX-100-PBS-BSA3, 15 min) and washed in 0.3% BSA 0.01% Tween 20 in PBS (PBS-BSA3-Tw). The eggs were then incubated 30 min in TRITC-conjugated *Lens culinaris* agglutinin (TRITC-LCA, Sigma, 25 µg/ml in PBS-BSA3-Tw), washed again in PBS-BSA3-Tw, stained with Hoechst and mounted. To detect the CG exudate, non-permeabilized eggs were incubated with 25 µg/ml TRITC-LCA for 15 min, then washed and fixed as described above. For actin staining, eggs were fixed and permeabilized with TX-100-PBS-BSA3 as previously described, washed, and incubated with FITC-conjugated phalloidin (66 nM, Invitrogen). After 30 min, the eggs were washed again, stained with Hoechst and mounted as described above.

### RT-PCR analysis

Total RNA from ZP-free eggs and cumulus cells was isolated using the RNAqueous-Micro kit (Ambion the RNA Company, TX, USA) following the manufacturer's instructions. Total RNA from liver, kidney, lung, testis and epididymis was isolated with Trizol® (Gibco BRL, Rockville, MD, USA), according to the manufacturer's recommendations. In all cases, first-strand cDNA was synthesized from total RNA (2 µg) using Moloney murine leukemia virus reverse transcriptase (MMLV-RT, Promega Co, Madison, USA) and oligo dT primers (Invitrogen) in the presence of a ribonuclease inhibitor. RNA was reverse transcribed and subjected to PCR using the primers and conditions described in Table 1. PCR products were resolved in 2% agarose gels and visualized by ethidium bromide staining. The identity of the amplified fragments was verified by DNA sequencing (Macrogen, Korea).

**Table 1:** RT-PCR primers and conditions.

mRNA	Primers	Conditions
*Plscr1*	Forward: 5′GCTACTGCAGCGCCACCAGC3′ Reverse: 5′CCCCAGGCCCACCTGGATGA3′	94°C, 5 min – 94°C, 1 min; 57°C, 1 min; 72°C, 1 min (×37) – 72°C, 1 min.
*Plscr2*	Forward: 5′AGCTGAGTCGCTGCTGGTGC3′ Reverse: 5′AACCATCTGCCCGAGGCTG3′	94°C, 5 min – 94°C, 1 min; 57°C, 1 min; 72°C, 1 min (×37) – 72°C, 1 min.
*Plscr3*	Forward: 5′GCTGAACGAGTGGAAACGTTCC3′ Reverse: 5′CACTGCTTGCTGATGCGGC3′	94°C, 5 min – 94°C, 1 min; 57°C, 1 min; 72°C, 1 min (×37) – 72°C, 1 min.
*Plscr4*	Forward: 5′GCTTCCCGCCACCAGCAGAG3′ Reverse: 5′ GGGCAGTTGGGCACAGGAGC3′	94°C, 5 min – 94°C, 1 min; 57°C, 1 min; 72°C, 1 min (×37) – 72°C, 1 min.
*Atp8a1*	Forward: 5′GCCTATGGGCAGAGCTCACAG3′ Reverse: 5′CGGATGGAGTGCGAACCAC3′	94°C, 5 min – 94°C, 1 min; 57°C, 1 min; 72°C, 1 min (×35) – 72°C, 1 min.
*Atp8a2*	Forward: 5′GGCCACGTCTGTTGGAGACC3′ Reverse: 5′GGCTCACTGGAGGAGGACAGG3′	94°C, 5 min – 94°C, 1 min; 60°C, 1 min; 72°C, 1 min (×40) – 72°C, 1 min.
*Cd52*	Forward: 5′GGCCTGCAGACTGTCCTGAACTC3′ Reverse: 5′GGAAACTGCAGGCACCCGCATC3′	94°C, 5 min – 94°C, 1 min; 60°C, 1 min; 72°C, 1 min (×30) – 72°C, 1 min.

### Calculations and Statistical Analysis

In all cases data represent the mean ± s.e.m. of at least 3 independent experiments. In each independent experiments between 6 and 15 eggs/group were evaluated. Statistical analyses were performed using the GraphPad Prism Software (San Diego, CA, USA). The fluorescence intensity values in fertilized eggs at different times and the percentages of labeled eggs were analyzed using one-way ANOVA and Holm-Sidak post-test. Results were considered significantly different at p<0.05

## Results

### PS exposure in fertilized eggs

**Figure 1:**
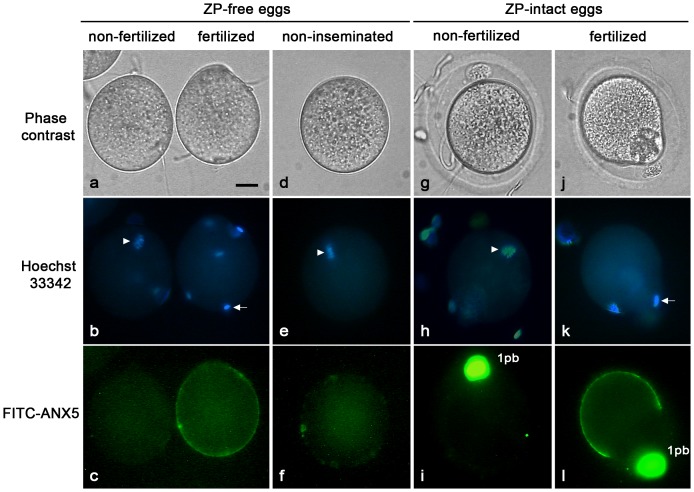
PS exposure in *in vitro* fertilized eggs. Phase contrast, Hoechst staining and FITC-ANX5 labeling of mouse eggs. ZP-free eggs were incubated with (a–c) or without (d–f) sperm in the presence of FITC-ANX5. After 1 h, eggs were stained with Hoechst 33342 for DNA visualization (b and e), and observed. Note the fluorescent labeling on a fertilized egg (c, right egg) and its absence in a non-fertilized egg present in the same fertilization drop (c, left egg) and in a non-inseminated egg (f). Also note the presence of bound sperm on both fertilized and non-fertilized eggs (a). ZP-intact eggs were inseminated and gametes co-incubated during 3 h (g–l). FITC-ANX5 was added during the last hour of gamete co-incubation. Eggs were then stained with Hoechst 33342 (h and k), and observed. Note the green fluorescent labeling on a fertilized egg (l), absent in a non-fertilized egg (i). In both cases, the first polar body (1 pb) is brightly fluorescent. Arrowheads: metaphase II chromosomes. Arrows: penetrating sperm head. Results are representative of 3 independent experiments in each of which 6–15 eggs/group were evaluated. Bar  = 20 µm.

To evaluate the exposure of PS in eggs following fertilization, ZP-free eggs were inseminated in the presence of FITC-conjugated Annexin V (FITC-ANX5), a protein that specifically binds PS, and observed without fixation after 1 h of gamete co-incubation. In parallel, non-inseminated ZP-free eggs were incubated with FITC-ANX5 and also observed. All fertilized eggs displayed a positive labeling whereas non-fertilized eggs present in the same fertilization drop (Fig. 1a–c) or non-inseminated eggs (Fig. 1d–f) were not labelled, a finding consistent with a previous report that PS is not exposed in ovulated non-inseminated mouse eggs [17]. Similar results were obtained when ZP-intact eggs were used, although a bright fluorescent labeling corresponding to the first polar body was observed in both fertilized and non-fertilized eggs (Fig. 1g-l). In all cases, PS labeling was localized over the entire surface of the egg with the exception of a negative area corresponding to the amicrovillar region overlying the meiotic spindle (Fig. 1b and c). It is interesting to note that the presence of FITC-ANX5 during gamete co-incubation had no effect on the percentage of fertilized eggs (data not shown), indicating that PS is exposed in fertilized eggs but is unlikely required for gamete fusion.

**Figure 2:**
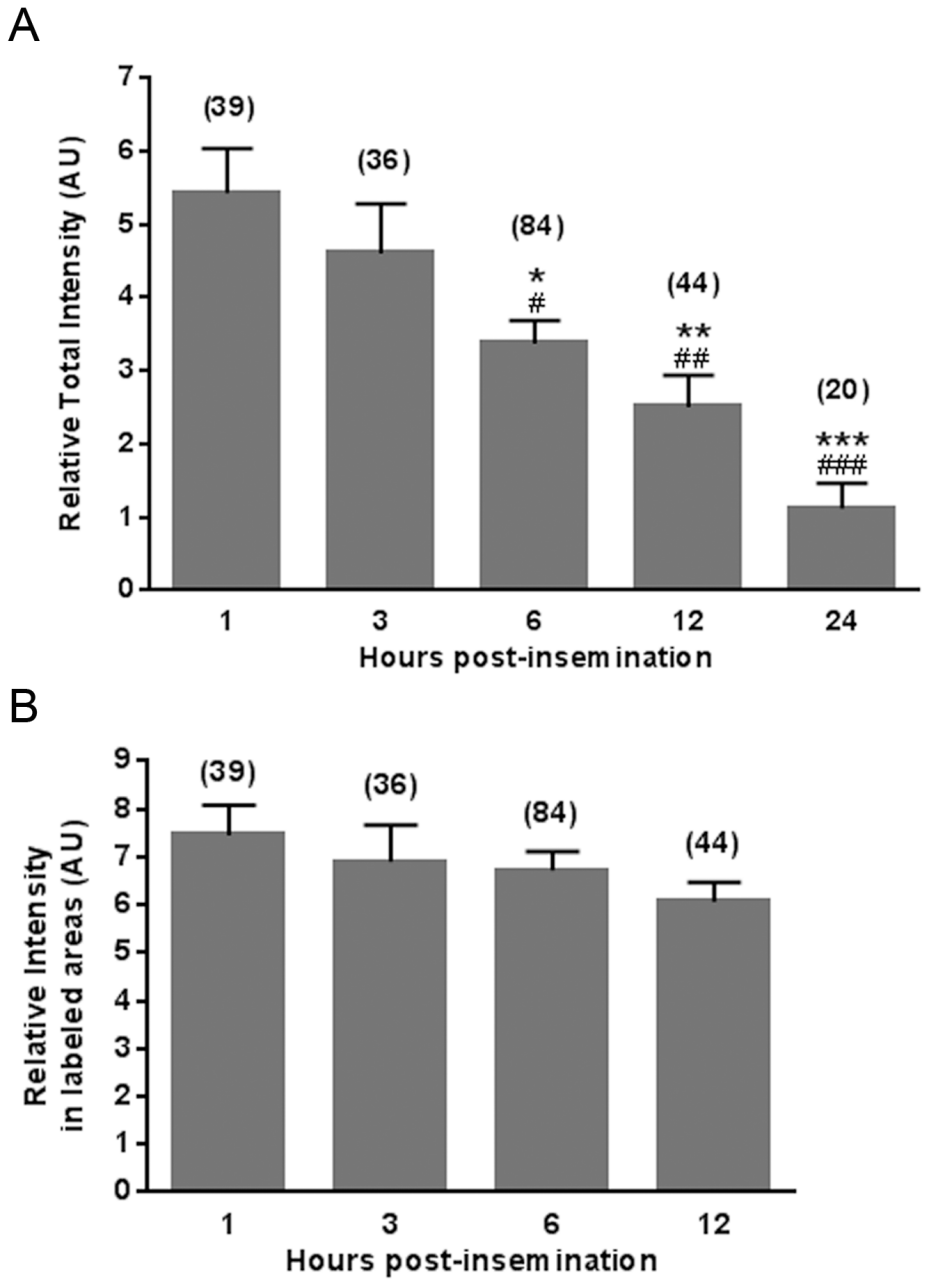
Quantification of exposed PS on the surface of fertilized eggs. ZP-free eggs were incubated in the presence or absence of sperm, and at different post-insemination (p.i.) times (1–24 h), they were stained with FITC-ANX5 and Hoechst 33342. Each egg was photographed, and the total fluorescent intensity/area for fertilized eggs relative to that measured in non-inseminated eggs (A) was calculated, as well as calculating the intensity within the labeled areas normalized to the intensity measured in non-labeled areas (B) using the ImageJ software. Each bar represents the mean value ± s.e.m. of at least 3 independent experiments in each of which 6–15 eggs/group were evaluated. The total number of analyzed eggs for each group is presented in brackets. # p<0.05 vs 1, 3 and 12 h; * p<0.001 vs 24 h; ## p<0.05 vs 6 and 24 h; ** p<0.001 vs 1 and 3 h; ### p<0.05 vs 12 hs; *** p<0.001 vs 1, 3 and 6 h.

To analyze the kinetics of PS exposure in fertilized eggs, FITC-ANX5 labeling of eggs was evaluated at different times post-insemination (p.i.). Although none of the non-fixed MII eggs exhibited fluorescent labeling at any of the evaluated times (1, 3, 6, 12 and 24 h p.i.), >85% of the non-fixed fertilized eggs were labeled up to 12 h p.i. At 24 h p.i., when the first mitotic division has already occurred, none of the 2-cell embryos displayed any staining, indicating that PS exposure was transient. Evaluation of fluorescence intensity in labeled eggs revealed that the total surface fluorescence intensity observed from 6 h p.i. on was less than that at 1 and 3 h p.i. (Fig. 2A). However, the fluorescence intensity in the labeled areas was similar along time (Fig. 2B), suggesting that the decrease in total fluorescence intensity was due to a decrease in the surface area exposing PS.

**Figure 3:**
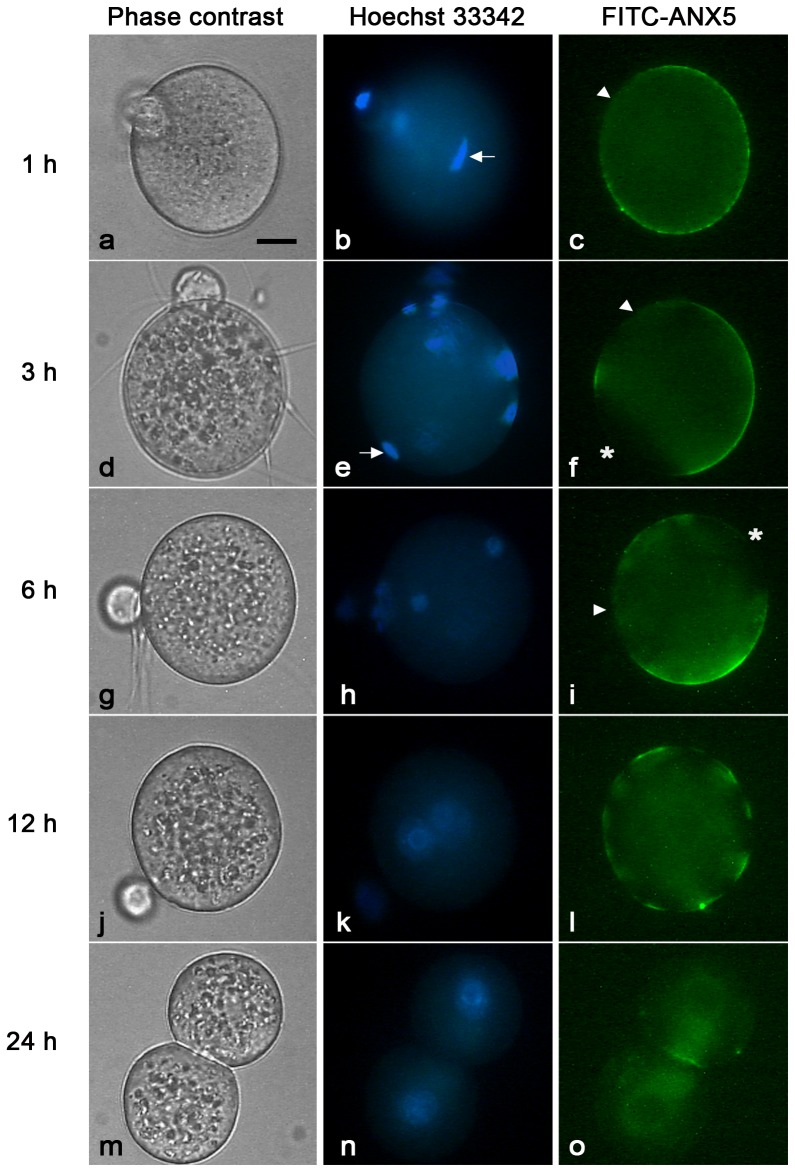
Localization of the fluorescent labeling observed at different times after insemination. Phase contrast, Hoechst staining and FITC-ANX5 labeling of fertilized eggs. ZP-free eggs were inseminated and gametes were co-incubated for 1 (a–c), 3 (d–f), 6 (g–i), 12 (j–l) and 24 h (m–o). FITC-ANX5 was added during the last hour of gamete co-incubation. Eggs were then stained with Hoechst 33342 for DNA visualization, and observed. Arrow: decondensed sperm head. Arrowhead: negative area corresponding to the membrane overlying the meiotic spindle. Asterisk: negative area corresponding to the membrane overlying the sperm entry site. Results are representative of 3 independent experiments in each of which 6–15 eggs/group were evaluated. Bar  = 20 µm.

**Figure 4:**
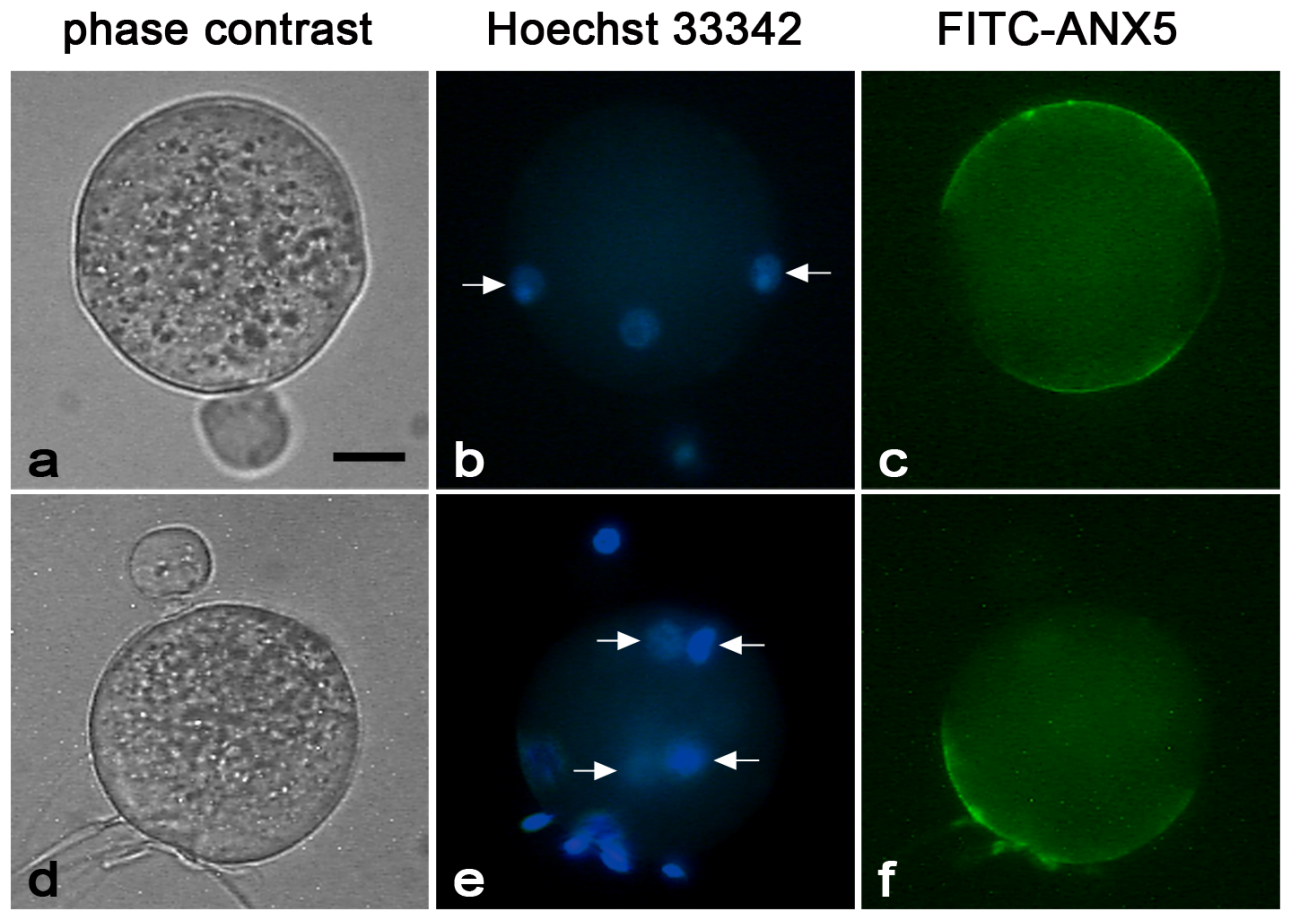
PS exposure in polyspermic eggs. Phase contrast, Hoescht staining, and FITC-ANX5 labeling of polyspermic eggs. ZP-free eggs were inseminated with a high sperm concentration, and the gametes were co-incubated for 3 h. FITC-ANX5 (c,f) was added during the last hour and eggs were then stained with Hoechst 33342 (b,e) to determine the number of penetrating sperm (arrows). Note the decrease in the PS exposing surface in an egg penetrated by 4 sperm (d–f) compared to that observed in an egg penetrated by 2 sperm (a–c). Results are representative of 3 independent experiments in each of which 6–15 eggs/group were evaluated. Bar  = 20 µm.

When the localization patterns were analyzed at different time points, we observed that at 1 h p.i. (Fig. 3a–c) labeling was over the entire surface of the egg, except for the region overlying the meiotic spindle. At 3 h p.i. (Fig. 3d–f), when most of the eggs contain a decondensing sperm head and have emitted the second polar body, the labeling pattern was similar but a second area devoid of staining was observed for the membrane overlying the decondensing sperm head. This pattern did not change at 6 h p.i. (Fig. 3g–i), when most of the eggs were at the two-pronucleus stage. At 12 h p.i. (Fig. 3j–l), isolated patches of fluorescence were observed, and no fluorescence was detected after the first mitotic division (Fig. 3m–o). Given that by 3 h after insemination, FITC-ANX5-labeling had disappeared from the region where the sperm entered the egg, the presence and localization of the negative areas in polyspermic eggs was analyzed. Results showed that eggs presented multiple or larger negative areas as the number of penetrating spermatozoa increased (Fig. 4), supporting the idea that incorporation and/or decondensation of sperm generated these negative areas.

**Figure 5:**
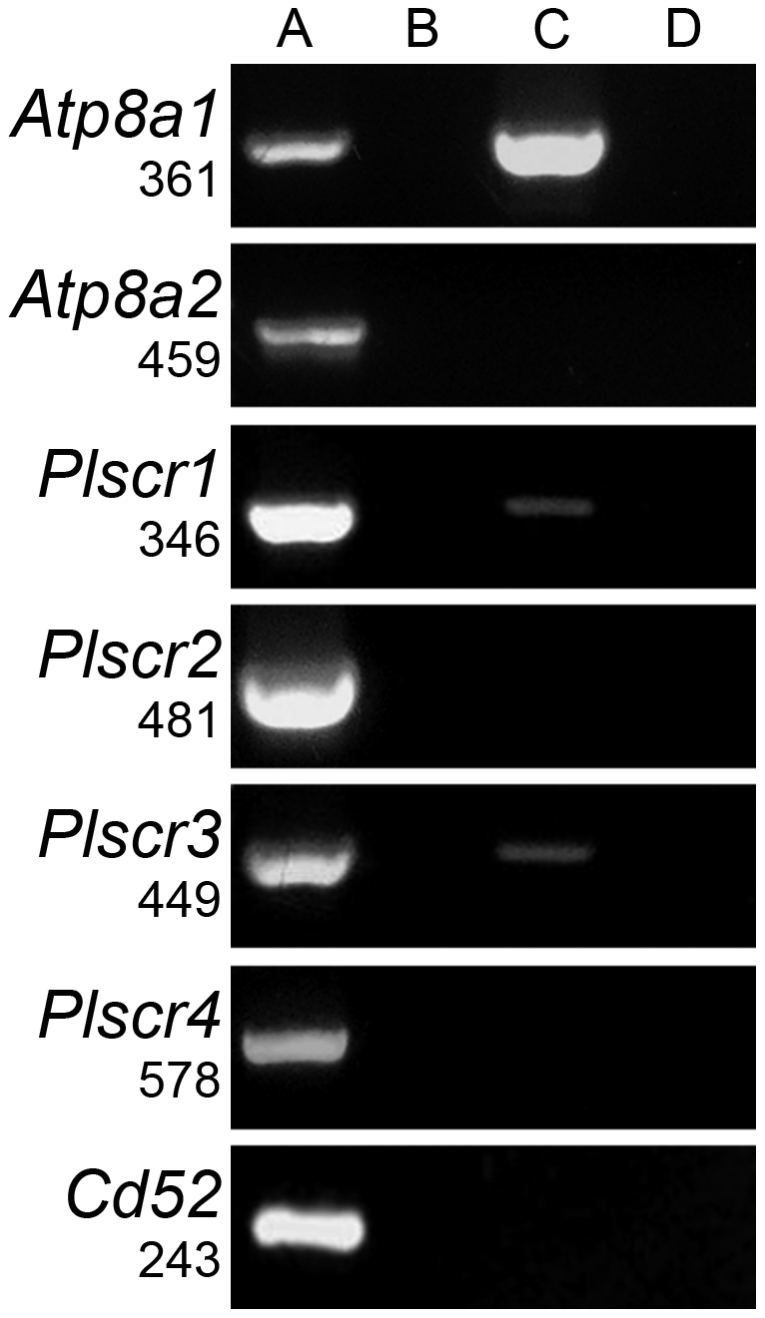
RT-PCR analysis of flippases and scramblases expression in mouse eggs. Total mouse egg RNA was subjected to RT-PCR using specific primers for each tested enzyme. Products were separated on 2% agarose gels and stained with ethidium bromide (lane **C**). As positive controls, total RNA from different tissues were used for each tested enzyme (lane **A**): epididymis for *Atp8a1*, testis for *ATP8a2*, liver for *Plscr1*, kidney for *Plscr2*, lung for *Plscr3* and *Plscr4*, and cumulus cells for *Cd52*. As negative controls, water was used as template for the reverse transcription (lane **B**) and during the amplification reaction (lane **D**). The expression of *Cd52* was evaluated to control contamination with cumulus cells. Results are representative of 3 independent egg RNA isolations.

The distribution of PS in the plasma membrane depends on the concerted action of flippases and scramblases. In mouse, ESTs for two flippases (*Atp8a1* and *Atp8a2*, Ref Seq., NM_001038999 and NM_015803, respectively) and four scramblases (*Plscr1*-*4*, Ref. Seq.: NM_011636.2, NM_001195084, NM_023564, NM_178711) have been detected in different tissues. RT-PCR analysis showed that whereas no amplification was obtained in eggs for *Atp8a2* and *Plscr2*, bands with the expected sizes for *Atp8a1*, *Plscr1* and *Plscr3* were detected (Fig. 5), whose identity was confirmed by DNA sequencing. These positive reactions could not be attributed to a contamination of the egg samples with cumulus cells because *Cd52*, a cumulus-specific gene [18], was not detected (Fig. 5). These results suggest that these scramblases and flippases are responsible for the observed PS mobilization.

### PS exposure in parthenogenetically activated eggs

**Figure 6:**
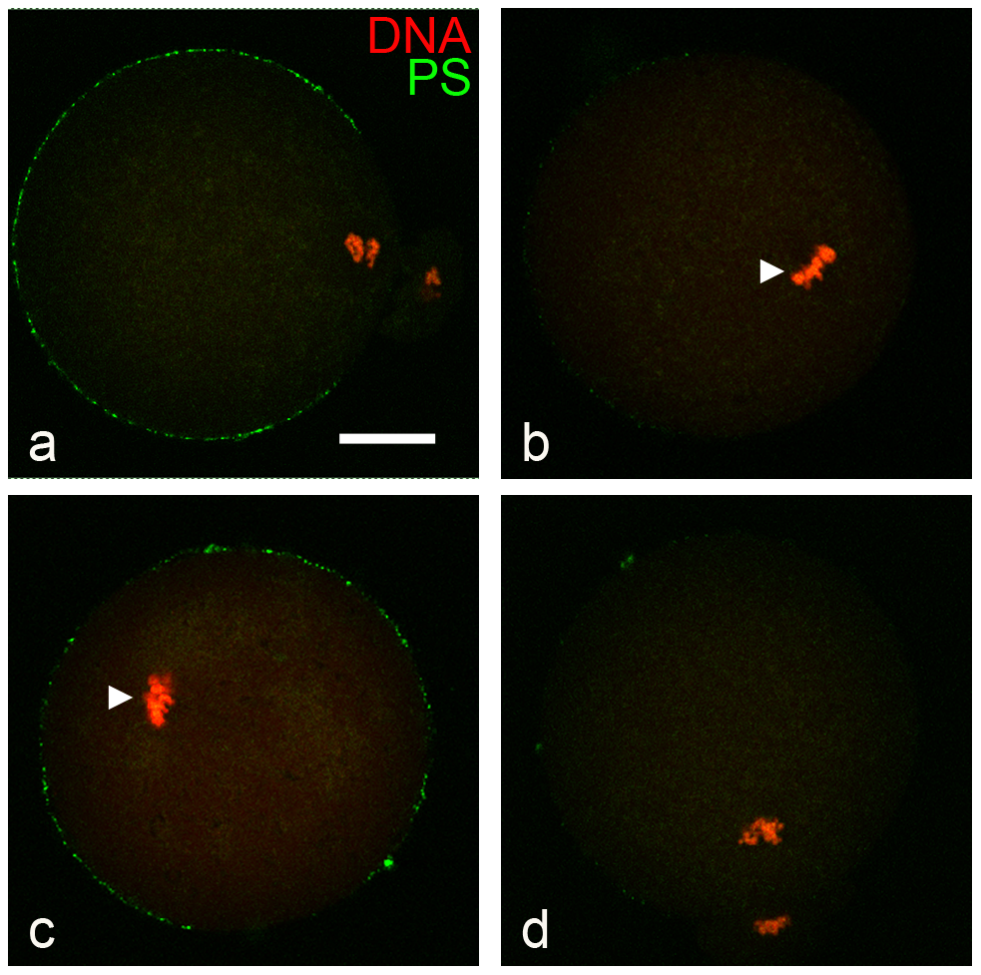
Exposure of PS in parthenogenetically activated eggs and Ca^2+^ requirement. Wild-type ZP-free eggs were incubated in the absence (a) or presence (b) of 50 µM BAPTA-AM prior to activation with 10 mM SrCl_2_. (c) Zona-free eggs recovered from CaMKIIγ^−/−^ females were activated with 10 mM SrCl_2_. (d) Wild-type ZP-free eggs were activated with 100 µM TPEN. In all cases, eggs were then incubated for an additional hour in the presence of FITC-ANX5 (green), fixed, and DNA was stained with TO-PRO3 (red) for confocal imaging. Arrowhead points metaphase II spindles. Note resumption of meiosis in (a) and (d), and exposed PS labeling in (a) and (c). Results are representative of 3 independent experiments in each of which 6–15 eggs/group were evaluated. Bar  = 20 µm.

Considering that the entry of the fertilizing sperm triggers egg activation, the exposure of PS that occurs after gamete fusion could be caused by sperm-egg fusion and uncoupled from egg activation or be a consequence of egg activation. To discriminate between these two possibilities, MII eggs were parthenogenetically activated with SrCl_2_, incubated with FITC-ANX5, fixed, and observed under a confocal microscope. As a control, 2-cell embryos were treated with SrCl_2_ and FITC-ANX5. Whereas all treated 2-cell embryos were negative (data not shown), 93% of the SrCl_2_-activated eggs presented a fluorescent labeling similar to that previously observed in fertilized eggs (Fig. 6a), indicating that the exposure of PS is induced by egg activation.

To determine whether PS exposure was linked to the increase in intracellular Ca^2+^ that occurs during fertilization/activation, eggs were incubated with the membrane-permeable Ca^2+^ chelator BAPTA-AM prior to SrCl_2_ activation. Under these conditions, neither meiosis resumption nor exposure of PS was observed (Fig. 6b). To ascertain whether the lack of PS externalization in this case was due to the absence of an increase in intracellular Ca^2+^ or to the lack of meiotic exit, eggs from CaMKIIγ^−/−^ mice, which display Ca^2+^ spikes but do not exit MII following fertilization [13], were activated with SrCl_2_ and incubated with FITC-ANX5. All the eggs contained MII spindles and an ANX5-fluorescent labeling similar to that observed in activated wild-type eggs (Fig. 6c). As an alternate approach, PS exposure was analyzed in eggs activated with TPEN, a Zn^2+^ chelator that induces MII exit without causing Ca^2+^ mobilization [19]. In this case, most eggs underwent MII exit but no fluorescent labeling for FITC-ANX5 was observed (Fig. 6d). Altogether, these results indicate that an increase in intracellular Ca^2+^ is required for PS exposure in eggs.

**Figure 7:**
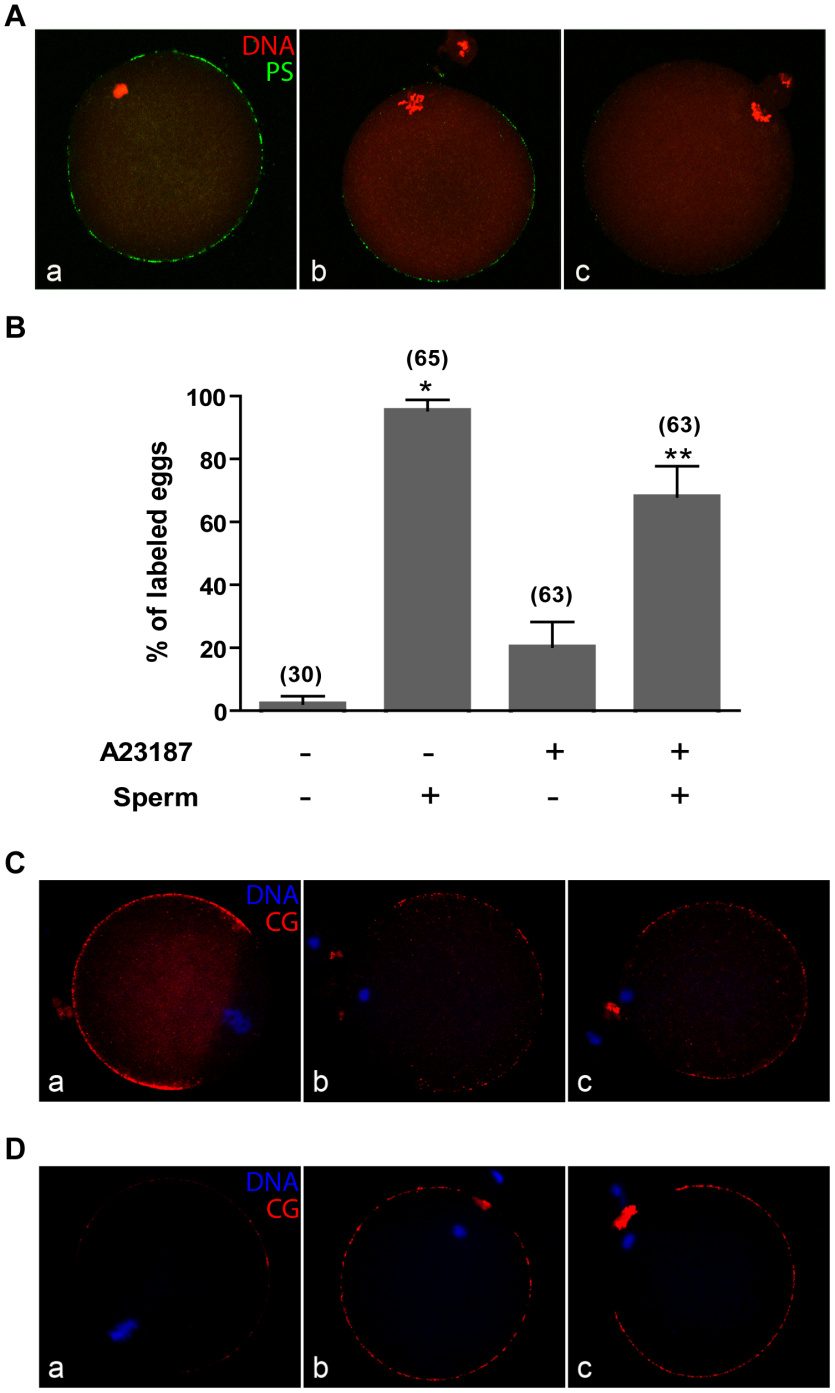
Exposure of PS in eggs incubated with different activating agents. (**A**) ZP-free eggs were parthenogenetically activated with either 10 mM SrCl_2_ (a), 7% ethanol (b) or 5 µM Ca^2+^ ionophore A23187 (c). FITC-ANX5 (green) was added during the last hour of incubation. Eggs were then fixed, and DNA was stained with TO-PRO 3 (red) for confocal imaging. Note resumption of meiosis in all cases, and exposed PS labeling only on SrCl_2_- and ethanol-activated eggs. Results are representative of 5 independent experiments in each of which 6–15 eggs/group were evaluated. (**B**) ZP-free eggs were incubated with 5 µM Ca^2+^ ionophore A23187. Those exhibiting a second polar body were inseminated, and the gametes co-incubated for 3 h. FITC-ANX5 was added during the last h of incubation, and eggs were then stained with Hoechst 33342 to evaluate the presence of penetrating sperm. The percentage of eggs presenting exposed PS was determined in each group. Bars represent the mean value ± s.e.m. of 5 independent experiments in each of which 6–15 eggs/group were evaluated. The total number of analyzed eggs in each group is presented in brackets. * p<0.001 vs control (non-inseminated non-activated) eggs; ** p<0.05 vs control and vs inseminated eggs. (**C** and **D**) ZP-free eggs were incubated in the absence (a) or presence (b) of SrCl_2_, or with 5 µM Ca^2+^ ionophore A23187 (c). (**C**) Eggs were fixed, permeabilized and stained for cortical granule (CG) content with TRITC-LCA (red). DNA was stained with Hoechst 33342 (blue). Note the decreased CG-labeling in SrCl_2_- and ionophore- activated eggs compared to control. (**D**) The cortical granules exudate was stained with TRITC-LCA (red). Eggs were then fixed and stained with Hoechst. Note the increase in CG exudate labeling in SrCl_2_- and ionophore- activated eggs compared to control. Results are representative of 3 independent experiments in each of which 6–15 eggs/group were evaluated. Bar  = 20 µm.

Although SrCl_2_ induces Ca^2+^ oscillations similar to those induced by the fertilizing sperm [20], both calcium ionophore and ethanol produce a single increase in [Ca^2+^]_i_ that is derived from intracellular or mainly extracellular sources, respectively [21], [22]. To evaluate further the role of Ca^2+^ in PS exposure, eggs were incubated either with 10 mM SrCl_2_, 7% ethanol or 5 µM Ca^2+^ ionophore A23187 which resulted in an incidence of activation of 94%, 97% and 73%, respectively. Whereas a high percentage of eggs activated with SrCl_2_ (96%) or ethanol (90%) were ANX5-positive, most (94%) of the Ca^2+^ ionophore-activated eggs were ANX5-negative (Fig. 7A). Similar results were obtained when different ionophore concentrations (1–5 µM) were used (data not shown). Interestingly, when Ca^2+^ ionophore-activated eggs were inseminated, FITC-ANX5 staining was observed for the fertilized eggs (Fig. 7B), which indicates that the eggs still retained the ability to externalize PS. These results suggest that it is the source of Ca^2+^, not just an increase in Ca^2+^, that is required to externalize PS.

It was formally possible that the membrane fusion events involved in cortical granule (CG) exocytosis stimulate PS exposure. Such a linkage does not exist because Ca^2+^ ionophore-activated eggs underwent CG exocytsis, as determined by a loss of LCA staining in permeabilized eggs or detection of the CG exudate in non-permeabilized eggs (Fig. 7C and D).

### PS externalization requires microfilaments

**Figure 8:**
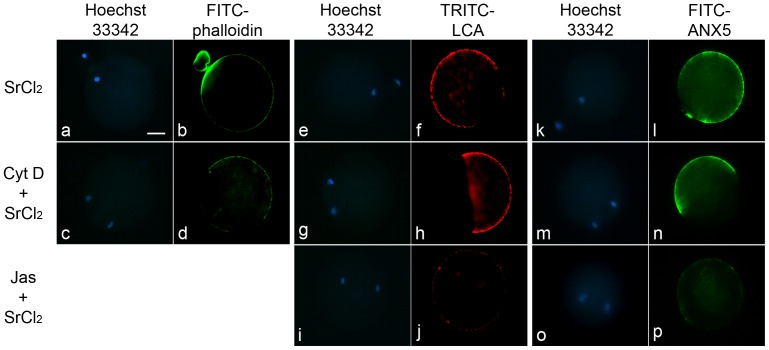
Effect of cytoskeleton perturbing drugs on activation-induced PS exposure. ZP-free eggs were incubated in medium alone, or medium containing 10 µM cytD or 0.5 µM Jas prior to activation with 10 mM SrCl_2_. In each case, eggs were then divided into three groups. One group was fixed, permeabilized, and stained with FITC-phalloidin for analyzing actin distribution (b,d). This staining was not performed for Jas-treated eggs. Another group was fixed, and cortical granule exudates detected by staining with TRITC-LCA (f, h, j). The third group of eggs was incubated with FITC-ANX5 for 1 h, and observed (l, n, p). In all cases, DNA was stained with Hoechst 33342 (a, c, e, g, i, k, m,o). Note resumption of meiosis without cytokinesis in cytD- and Jas-treated eggs, as well as a decrease in CG exocytosis and PS exposure in Jas-treated eggs. Results are representative of 3 independent experiments in each of which 6–15 eggs/group were evaluated. Bar  = 20 µm.

To establish whether the cytoskeleton modifications that take place in the egg after fertilization/activation [23] influenced exposure of PS, the role of the actin cytoskeleton was examined by treating eggs with cytochalasin D (cytD) or jasplakinolide (Jas) prior to activation with SrCl_2_ and incubation with FITC-ANX5. inhibits addition of monomeric G-actin to microfilaments and reduces actin polymerization [24], whereas Jas induces actin polymerization and stabilizes existing actin microfilaments [25]. The effectiveness of each treatment was monitored by staining a group of eggs in parallel with either FITC-phalloidin to detect actin microfilaments or TRITC-LCA to detect CG exudates. Actin staining was not performed in Jas-treated eggs because Jas competitively inhibits the binding of phalloidin to actin [25]. Treatment of non-activated eggs with 10 µM cytD or 0.5 µM Jas did not induce MII exit, CG exocytosis or exposure of PS (data not shown). As expected, cytD-treated activated eggs showed an abnormal distribution of actin (Fig. 8a–d), displayed a CG exudate similar to controls (Fig. 8e–h), and failed to emit the second polar body (Fig. 8c, g and m). Jas-treated activated eggs did not emit the second polar body (Fig. 8i and o) and CG exocytosis did not occur (Fig. 8j). Whereas FITC-ANX5 staining in cytD-treated activated eggs was similar to that of control eggs, Jas-treated activated eggs showed a decreased staining (Fig. 8p), supporting that PS externalization requires both egg activation and microfilament depolymerization.

## Discussion

We demonstrate here that fertilization/egg activation of mouse eggs is accompanied by externalization of PS that does not lead to apoptosis. The appearance of PS on the surface of apoptotic cells identifies them as targets for engulfment by phagocytic cells that directly recognize exposed PS through Tim4 receptor [3],[26],[27]. Such engulfment could not occur for fertilized/activated eggs because the *zona pellucida* would provide a physical barrier for such an interaction. Although the functional consequences of PS externalization we observe are not known, a requirement of PS exposure in cellular processes different from apoptosis has been demonstrated in recent years.

The PS exposure is transient and external PS is not detected in 2-cell embryos, with loss of external PS occurring sometime between the late 1-cell stage and shortly after cleavage to the 2-cell stage. Because total fluorescence intensity decreases from the pronuclear stage, disappearance of PS from the external leaflet may be coupled with cessation of Ca^2+^ oscillations that occurs with pronucleus formation [28]. If PS externalization has a physiological function as a consequence of fertilization/egg activation, it would have to be initiated during this window of external exposure. It is interesting to note that by 3 h post-insemination, PS labeling has disappeared from the sperm entry region. This loss of external PS could be attributed to the internalization or degradation of PS in that membrane region as a consequence of sperm entry or, alternatively, to incorporation of the sperm membrane into the egg plasma membrane, which has been observed in different species [29]. The finding that polyspermic eggs display multiple or larger negative areas and that parthenogenetically activated eggs do not show this negative area (Figs. 6 and 7) supports the second possibility.

Externalization of PS does not require resumption of meiosis but does require an increase in [Ca^2+^]_i_. PS externalization occurs in SrCl_2_-activated CaMKIIγ null eggs, which do not exit metaphase II arrest. In contrast, TPEN-activated eggs, which do not externalize PS, resume meiosis in the absence of an increase in [Ca^2+^]_i_. It is interesting to note that Ca^2+^ ionophore-activated eggs do not expose PS although CG exocytosis and MII exit occur. Sperm and SrCl_2_ induce oscillations in [Ca^2+^]_i_ and PS externalization. In contrast, ethanol and Ca^2+^ ionophore produce a single increase in [Ca^2+^]_i_ [21], but PS externalization is only observed following ethanol treatment. Therefore, the absence of PS exposure in ionophore-activated eggs cannot be attributed to the number of Ca^2+^ oscillations but rather to either the concentration of Ca^2+^ achieved during the cytoplasmic Ca^2+^ increase or the duration of this increase, which may differ from that induced by ethanol. It should also be noted that ethanol activation is induced in a complete medium whereas Ca^2+^ ionophore activation is induced in a Ca^2+^-free medium. In this regard, it has been recently shown that external Ca^2+^ entry is needed for complete egg activation [30], supporting the idea that the source of Ca^2+^ is important for PS exposure. Because inseminated ionophore-activated eggs are able to expose PS other factors may also be involved in PS exposure.

Successful pregnancy and childbirth have been reported after the use of Ca^2+^ ionophore to activate eggs in cases of fertilization failure after ICSI [31], [32]. Our results show, however, that ionophore-induced activation does not completely mimic the activation produced by sperm. Similarly, translocation of PKC-alpha to the plasma membrane that normally occurs following fertilization of rat eggs does not occur when rat eggs are activated by the Ca^2+^ ionophore ionomycin [33]. These findings raise concern about use of such approaches in the treatment of human infertility and also indicate that exposure of PS could be a useful tool to evaluate differences among egg-activation treatments.

The actin cytoskeleton plays a role in regulating PS exposure in different cell types [34], [35]. Although neither cytD nor Jas treatment promoted PS externalization, Jas, but not cytD, inhibited PS externalization when the eggs were activated. Thus, in addition to a role for an increase in [Ca^2+^]_i_ in PS externalization, like other cell types, remodelling of the actin cytoskeleton is also implicated.

*Atp8a1* flippase and scramblases *Plscr1* and *3* are expressed in eggs. The presence of *Atp8a2* ESTs has been reported for mouse oocytes (http://www.ncbi.nlm.nih.gov/UniGene/ESTProfileViewer.cgi?uglist=Mm.319599), but we failed to detect *Atp8a2* transcripts by RT-PCR in eggs. This difference may be due to the sensitivity of the RT-PCR method and relative abundance of the *Atp8a2* transcript. Interestingly, an increase in [Ca^2+^]_i_ activates scramblases and inhibits flippases [11], and this mechanism induces PS exposure in different cell types [36], [37], [10]. A similar mechanism could operate in eggs because PS exposure requires an increase in [Ca^2+^]_i_. Nevertheless, because calcium ionophore does not induce PS exposure, a role for other factors also in regulating scramblase and flippase activity cannot be excluded. Whereas PLSCR3 is mainly localized to mitochondria [10], PLSCR1, one of the best characterized scramblases, can interact with several cytoplasmic protein kinases, including c-Abl and members of the Src family [38], [39] that play critical roles during egg activation in invertebrates and mammals, respectively [40], [41], [42]. In MII eggs, tyrosine phosphorylated proteins are homogeneously distributed in the cytoplasm whereas in fertilized eggs these proteins accumulate in the egg cortex up to the pronucleous stage [43]. The coincidence between change in distribution and the PS exposure pattern coupled with scramblases being phosphorylated by c-Abl and Src, suggest a possible involvement of these kinases in regulating PS exposure in eggs.

The mobilization of PS in fertilized/activated eggs could simply be a consequence of the increase in [Ca^2+^]_i_ that occurs during the course of fertilization but not play any physiological role. The appearance of PS in the external leaflet of the egg membrane, however, could be involved in establishing the membrane block to polyspermy. Because PS is negatively charged, its externalization could affect the net charge of the membrane [44] and prevent binding of supernumerary sperm. The situation, however, is likely more complex because cytochalasin D leads to an increase in polyspermy [45] but it does not affect PS exposure, and activated eggs do not mount a polyspermy response [46],[47] but do externalize PS, i.e., PS exposure is not solely responsible to establish the membrane block to polyspermy. Alternatively, when located on the inner leaflet of the plasma membrane, PS could serve as a molecular anchor for proteins containing the PS-binding domain C2 [48], [49] and thereby by regulating their subcellular localization and/availability modulate their activity. In this regard, several proteins postulated to participate in egg activation contain C2 domains. For example, the sperm-specific PLCζ [50], [51] appears to trigger Ca^2+^ oscillations through the production of IP_3_ [52]. Conventional and novel PKCs also possess a C2 domain and their translocation to the plasma membrane following egg activation requires a C2 domain [53], [54]. It is possible, therefore, that PS mobilization in eggs regulates the activity of PLCζ and/or PKCs during egg activation. In addition, the negatively charged PS promotes plasma membrane recruitment of positively charged proteins such as MARCKS and vinculin [55], [56], [57], both proposed to be involved in fertilization-associated events [58]. In summary, our results show for the first time the existence of a transient exposure of PS in fertilized eggs not associated with apoptosis and induced by the Ca^2+^ rise produced during egg activation. The functional relevance of the transient exposure of PS for egg activation is currently under investigation.
